# Characterization of histone acetyltransferases and deacetylases and their roles in response to dehydration stress in *Pyropia yezoensis* (Rhodophyta)

**DOI:** 10.3389/fpls.2023.1133021

**Published:** 2023-05-16

**Authors:** Zehao Zhang, Huijuan Qian, Zhongshi Wang, Ying Pang, Xiaowei Guan, Ansgar Poetsch, Dongmei Wang

**Affiliations:** ^1^ Key Laboratory of Marine Genetics and Breeding (OUC), Ministry of Education, Qingdao, China; ^2^ College of Marine Life Sciences, Ocean University of China, Qingdao, China; ^3^ Department of Plant Biochemistry, Ruhr University Bochum, Bochum, North Rhine-Westphalia, Germany

**Keywords:** *Pyropia*, histone acetylation, histone acetyltransferase, histone deacatylases, dehydration (drought stress), red algae

## Abstract

Histone acetylation is one of the most pivotal epigenetic mechanisms in eukaryotes and has been tightly linked to the regulation of various genes controlling growth, development and response to environmental stresses in both animals and plants. Till date, the association of histone acetylation to dehydration stress in red algae and genes encoding the enzymes responsible for histone acetylation: histone acetyltransferases (HATs) or histone deacetylases (HDACs), remains largely unknown. In this study, in silico analysis of the red seaweed *Pyropia yezoensis* identified 6 HAT genes and 10 HDAC genes. These genes displayed good synteny in genome loci with their *Pyropia haitanensis* orthologs except for a putative gene duplication event in HDAC and a loss of one HAT gene in *P. yezoensis*. According to the conserved domains and phylogenetic analysis, they encoded three GCNA5-, one TAFII250- and one MYST-HAT, as well as five HDA1-and five SIRT-HDACs. The sirtuin-domain of Py06502 harbored a ~100 aa insert and interestingly, this insertion was specifically observed in Bangiales species. Two nuclear-localized HATs were transcriptionally up-regulated at the early stage of dehydration and so were two nuclear HDA1s when moderate dehydration started, suggesting their potential roles in modulating downstream gene expression to facilitate dehydration adaptation by changing histone acetylation patterns on relevant regulatory elements. This was experimentally confirmed by the increased decline in photosynthesis efficiency during dehydration when HAT and HDAC activities were inhibited by SAHA and MB-3, respectively. Transcriptional patterns of multiple dehydration-responsive genes after water loss were strongly affected by MB-3 or SAHA treatment. This study provides the first insight into the regulation and function of HAT/HDAC during stress adaptation in red algae.

## 1 Introduction

Eukaryotic nuclear DNA is organized into a DNA-protein complex called chromatin. The nucleosome, comprised by 146 bp of DNA tightly surrounding an octamer of four histone proteins (H3, H4, H2A, and H2B), is the basic structural unit of chromatin (Vergara and Gutierrez, 2017). The affinity between DNA and histones in nucleosome hinders the recognition and binding of transcriptional regulators to the DNA locus, thereby repressing the initiation of gene expression (Chen and Tian, 2007). The acetylation of lysine residues on the amino-terminal tails of histone proteins neutralizes the charges and dramatically reduces the affinity of the tail for DNA (Shen et al., 2015). The resulting loosening of the histone complex from the DNA makes the DNA region accessible to the transcription machinery (Pfluger and Wagner, 2007). Thus, histone acetylation is an important epigenetic mechanism in modulating gene expression and plays vital roles in growth, development and response to biotic and abiotic stresses in animals and plants.

Histone acetylation and deacetylation are catalyzed by histone acetyltransferase (HAT) and histone deacetylase (HDAC) (Kuo and Allis, 1998) respectively. So far, genome-wide identification of HAT and HDAC genes has been achieved in many animal and plant species (Chu and Chen, 2018; Fellous et al., 2019). HATs are subdivided into four families: the general non-depressible GCN5-related N-terminal acetyltransferase (GNAT), the MYST family consisting of MOZ, YBF2, SAS3 and TIP60, the CREB (cAMP-responsive element-binding protein)-binding protein family (CBP), as well as the TATA-binding protein-associated factor 1 (TAFII250) family. HDACs in eukaryotes are usually organized into two main groups based on the domain composition: the yeast-reduced potassium dependency 3 RPD3/HDA1 superfamily and the Sirtuins (SIRT) family (Pandey et al., 2002). The SIRT family was divided into five subfamilies: class I, class II, class III, class IV and class U. Class U exists in several firmicute (gram positive) bacteria and *Thermotoga maritima* with sirtuin gene sequences that seem intermediate between classes II and III and the classes I and IV. Classes I, II, III, and IV occur across a wide range of organisms, including prokaryotes, fungi, algae, plants and animals (Frye, 2000). Plants and some streptophyte green algae also express an additional plant-specific HDAC family, termed type-2 HDACs (HD2) (Bourque et al., 2016).

A wealth of studies revealed that *HAT*s and *HDAC*s are involved in acclimating environmental abiotic stresses, by reforming the chromatin structure and directing transcriptional changes. GCN5 was first characterized in corn and its expression increased under salt stress conditions (Zheng et al., 2019). Transcription of four *HAT*s in rice was significantly induced by drought stress and hyperacetylated lysine residues of histone H3 (Fang et al., 2014). Drought stress also induces different expression patterns of *HDAC* genes, resulting in changes in the levels of histone acetylation of drought response genes. In some plants, histone deacetylase HDA6 was found to be the ON/OFF switch of an essential drought-responsive network to stimulate the jasmonate (JA) signaling pathway to confer drought tolerance (Kim et al., 2017).

As a founding group of photosynthetic eukaryotes (Archaeplastida), red algae donate the plastids that form numerous other photosynthetic lineages, including the major primary producers in marine ecosystems (Ball et al., 2011). Thus, red algal cell biology has contributed dramatically to broader eukaryotic evolution and diversity. However, current knowledge of epigenetic mechanisms in stress adaptation is mainly from studies in green-lineage plants; little is known in red algae. *Pyropia yezoensis* and *Pyropia haitanensis*, belonging to the Bangiales, Rhodophyta, family are the two main marine-cultivated *Pyropia* species in nori farming (Yang et al., 2020). The thalli inhabit the upper intertidal zone and experience broad and extreme environmental stresses (dehydration, high temperature, high ultraviolet irradiance etc.) due to frequent emersion in tide flow(Zou, 2002; Huan et al., 2018). Their thalli can lose more than 80% of their water content in low tide and rapidly recover photosynthesis in rehydration. Therefor *Pyropia* species are of interest as red algal model organisms for studying molecular mechanisms in stress resistance. Recent studies have demonstrated extensive transcriptional changes in response to stresses in *Pyropia* species (Wang et al., 2019). It’s essential to understand the epigenetic factors that regulate gene expression and thereby boost the robustness under stress. Researchers found orthologous genes of components of SNF/SWI chromatin remodeling complex in *Pyropia* and other red algae, implicating the contributions of reforming chromatin structure in modulating gene expression (Stiller et al., 2018). The development of ChIP-Seq method and identification of a histone mark H3K9 in generation-specific genes further emphasized the essential roles of histone acetylation in regulating gene expression in *P. yezoensis* (Ueda et al., 2022). However, little is known about the key players responsible for adding or removing acetyl groups from histones to medicate chromatin structural changes. In this study, we identified genes encoding HAT and HDAC in *P. yezoensis* and *P. haitanensis* genomes *via* a bioinformatic approach. We also analyzed gene structure, synteny in genome location, conserved domains, subcellular localization and transcriptional variations in HAT and HDAC genes. Furthermore, we investigated the photosynthetic performance of *P. yezoensis* thalli under dehydration and transcriptional variations of some dehydration-responsive genes when the HAT and HDAC activities were inhibited.

## 2 Materials and methods

### 2.1 Algal cultivation

The pure line RZ of *P. yezoensis*, established by clonal cultivation of an isolated single somatic cell, was used for the experiments. Fresh leafy gametophytes were cultured in boiled natural seawater with Provasoli’s enrichment solution medium (PES) at 10°C in incubator, with a photon flux of 20 μmol photons·m^−2^·s^−1^ and a 12/12-h light/dark cycle. The medium was refreshed every three days. To obtain thalli with different water content, the absolute water content (AWC) of thalli was calculated using the fresh weight and its dry weight according to the methods described by Kim et al. (Kim et al., 2009). Thalli under normal growth conditions were harvested as the control group (AWC100). Before dehydration, the surface water of the thalli was removed by paper towels, and then the thalli were exposed to air for dehydration under 20 μmol photons m^−2^·s^−1^ at 8 ± 1°C. The thalli samples were collected until the total water content decreased by 30% (AWC70), 50% (AWC50), and 70% (AWC30). Samples were collected and placed in liquid nitrogen before gene expression analysis.

### 2.2 Genome−wide identification of HDACs and HATs in *P. yezoensis*

The *P. yezoensis* genome and protein sequences were obtained from our previous work (Wang et al., 2020). To identify candidate HDAC protein sequences in *P. yezoensis*, the Hidden Markov model (HMM) of the HDAC domains were downloaded from Pfam (http://pfam-legacy.xfam.org/, Pfam : PF00850) and used in HMMER (https://www.ebi.ac.uk/Tools/hmmer, e-value<1e^-5^). Protein sequences of *Arabidopsis thaliana* HAT (AtHAT) were downloaded from the Ensemble Plants (http://plants.ensembl.org/index.html) database (Bolser et al., 2015) and used as query references in blastp to identify HAT proteins in *P. yezoensis* (Camacho et al., 2009). General information of HDAC and HAT genes and proteins in *P. yezoensis* are shown in **Table 1**. Genome and protein sequences of other red algae were downloaded from the National Center for Biotechnology information (https://www.ncbi.nlm.nih.gov/). Synteny analysis between *P. yezoensis* and *P. haitanensis* genes was performed with RIdeogram (Hao et al., 2020). Gene structures of HATs and HDACs were visualized in TBtools (Chen et al., 2020). The functional domain analysis was performed using InterProScan and SMART (Blum et al., 2021; Letunic et al., 2021).

**Table 1:** General information of HDAC and HAT genes in *P. yezoensis*.

Class		Gene Name	Gene ID	Protein Sequence Length	Molecular Weight
HAT	GNAT	PyGCN5	*Py03649*	540	58007.48
		PyGNAT	*Py09866*	557	61941.84
		PyGNAT-like	*Py02283*	314	31359.41
	unclassified	PyHAT-like	*Py04694*	416	43044.2
	MYST	PyMYST	*Py00356*	915	94335.8
	TAF	PyTAFII250	*Py07125*	2297	223147.74
HDAC	HDA1-Class I	PyHDAC1	*Py10301*	538	57952.66
		PyHDAC2	*Py03239*	1134	111170.83
	HDA1-Class II	PyHDAC3	*Py04715*	550	54990.3
	HDA1-Class IV	PyHDAC4	*Py09197*	267	26619.29
		PyHDAC5	*Py04721*	301	29699.74
	SIRT- Class I	PySIRT1	*Py08944*	528	51029.41
		PySIRT2	*Py02258*	636	66419.99
	SIRT- Class IV	PySIRT3	*Py06502*	535	52880.38
		PySIRT4	*Py07153*	380	39303.39
	SIRT-Class II	PySIRT5	*Py01658*	367	35644.21

### 2.3 Multiple sequence alignment and phylogenetic analysis

Homologous HATs and HDACs in red algal species (*Porphyra umbilicalis*, *Porphyridium purpureum*, *Chondrus crispus*) and classic model organisms (*Arabidopsis thaliana* and *Saccharomyces cerevisiae*) were identified through BLASTP and used for phylogenetic analysis (Camacho et al., 2009). To show difference in sub-family of the SIRT family, we included homologs of *Homo sapiens* in the phylogenetic analysis of the SIRT family. Multiple alignment and phylogenies of HATs and HDACs were constructed using the maximal likelihood algorithm in MEGA7 with a bootstrap test (1000 replicates) (Kumar et al., 2016).

### 2.4 Subcellular localization analysis of PyHDACs and PyHATs

The full-length CDS without the termination codon of four HDACs and two HATs were cloned into the modified PCAMBIA1300 vector, under the control of the CaMV 35S promoter. Primers for PCR amplification of full-length CDS are listed in the **Table S1**. Each CDS was frame-fused to the green fluorescent protein (GFP) coding sequence. A vector expressing the GFP gene was used as the control. The resulting constructs, PyGNAT-like-Py02283:GFP, PyMYST-Py00356:GFP, PyHDAC1-Py10301:GFP, PyHDAC3-Py04715:GFP and PySIRT4-Py07153:GFP, were each transformed into Agrobacterium tumefaciens EHA105 and then introduced into Nicotiana benthamiana using an injection method as previously reported (Sparkes et al., 2006). The injected tobacco plants were cultured under low light for 2 days. The PySIRT1-Py08944 construct and its control were transformed into *Arabidopsis* mesophyll protoplasts (Yoo et al., 2007). Protein expression in the tobacco leaves was observed under an Olympus FV-1000 microscope (Olympus, Japan). The excitation and emission wavelengths of the GFP signal were 488 nm and 510 nm, respectively, and those of the Chl signal were 640 nm and 675 nm, respectively.

### 2.5 RNA isolation and qRT−PCR analysis

Total RNA was extracted with RNeasy Plant Mini Kit (OMEGA) according to the manufacturer’s instructions. Next, first-strand cDNA was synthesized with 1 μg of total RNA using the HiScript III RT Super Mix for qPCR (+gDNA Wiper) kit (Vazyme Biotech). The gene expression levels of ubiquitin (UBC) were used as an internal control, and the relative gene expression values were calculated from 2^-△△Ct^ method. The sequences of the primers used are listed in **Table S1**.

### 2.6 SAHA and MB-3 treatment for *P. yezoensis* thalli

The 0.1mM stock solutions of SAHA (SML0061-25MG Sigma) and MB-3 (M2449-5MG Sigma) were prepared in DMSO and added to the culture medium of *P. yezoensis* thalli, respectively, to reach a final concentration of 0.1μM, according to a previous study in *P. yezoensis* by Guan et al. (Guan et al., 2022). Equivalent volumes of DMSO were added to control thalli culture. Three separate cultures were prepared for each treatment. 24 h later, thalli were collected for dehydration treatment as described above. Thalli of 100%, 70%, 50%, 30% water content were subjected to photosynthetic parameters measurement on Flour-Cam (Beijing Ecotech-Technology co. LTD). For each assay, nine individual thalli, every three from one individual culture of SAHA-treated, MB-3 treated, or DMSO control, were used as replications.

### 2.7 Data availability

The CDS sequence of HATs and HDACs of *P. yezoensis* and *P. haitanensis* used in this study can be found in the National Center for Biotechnology Information GenBank with accession number: OQ621440-OQ621441, OQ621447, OQ621504, OQ656315, OQ656318, OQ656322-OQ656326, OQ656370-OQ656374, OQ678374-OQ678388, OQ683344.

## 3 Results and discussion

### 3.1 Identification of the *HAT* and *HDAC* genes in *P. yezoensis*

**Figure 1:**
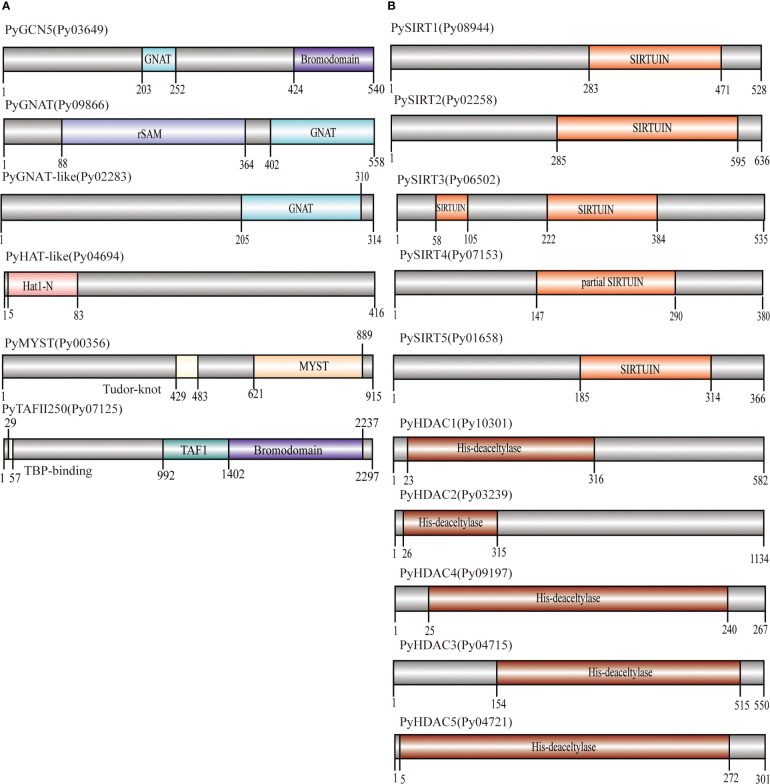
Domain architecture of PyHATs **(A)** and PyHDACs **(B)**. The names and protein lengths of the PyHATs and PyHDACs are indicated. Different domains are represented by different colors and lengths at their precise positions in the protein sequence from the N-terminus to the C-terminus.

We successively identified six *HAT* and 10 *HDAC* genes in the *P. yezoensis* genome through genome-wide homology searching. They encoded protein sequences ranged from 267 amino acids to 2297 amino acids, and the molecular weights ranged from 26619.29 to 223147.74 Da (**Table 1**). To further study the characteristic regions of the *P. yezoensis HAT* and *HDAC* genes, we analyzed the conserved domains of the proteins they encoded (**Figure 1A**). In plants, the GNAT family of HAT comprises three distinct sub-families denominated GCN5, HAT1, and ELP3 (Servet et al., 2010). Py03649 contains a C-terminal bromodomain and a GNAT-type HAT domain in the middle, showing the same domain architecture as plant GCN5 proteins (Aquea et al., 2017). Moreover, the protein identity is up to 41.25% between its GNAT-type HAT domain and *Arabidopsis* GCN5 (AtHAG1) (Benhamed et al., 2006; Benhamed et al., 2008). Therefore, Py03649 corresponds to the GCN5 protein in *P. yezoensis* (named as PyGCN5-Py03649 afterwards) and might be responsible for the acetylation of H3K14 and play similar regulatory roles as its *Arabidopsis thaliana* homolog in development and stress responses (Earley et al., 2007). Py09866 harbored a rSAM domain in addition to a C-terminal GNAT domain; therefore, it was annotated as the Elp3 subfamily in GNAT-type HAT in *P. yezoensis* (**Figure 1A**). C-terminal GNAT domain was also observed on Py02283 (**Figure 1A**). However, since this is the sole conserved domain and the protein identity with the *Arabidopsis* GNAT domain is lower than 25%, we cannot clearly designate it as a GNAT-family and annotated it as GNAT-like. Py04694 possesses a HAT1 domain at the N-terminus (**Figure 1A**). When blasting against the *Arabidopsis* genome, its best hit is the AtHAT1, albeit with protein identity lower than 30%. Moreover, it lacks the embedded GNAT-domain in the HAT1 domain, which is a typical architecture in AtHAT1. Although this absence of GNAT-domain is also found in rice HAT1 (OsHAG704), the protein identity between Py04694 and rice HAT1 is also too low to deduce a convincing category for Py04694 (Liu et al., 2012); further phylogeny analysis is required.

The MYST family is the largest and most divergent HAT family in animals, albeit not in plants. Two MYST proteins were found in *Arabidopsis* and function in gametophyte development (Latrasse et al., 2008). In *P. yezoensis*, only Py00356 was identified to have a C-terminal MYST-type HAT catalytic domain and a central tudor-knot domain, thus named PyMYST (**Figure 1A**). Py07125 has a C-terminal bromodomain, a central TAF1 domain and a TBP-binding domain at the N-terminus; therefore, it was categorized in the TAFII250-family HAT (**Figure 1A**). The CBP family is the most abundant HAT family in plants. Five genes are encoding CBP-HAT in *Arabidopsis* and are involved in the ethylene signaling pathway in plant development (Hinckley et al., 2019; Guo et al., 2021). However, we did not obtain any BLAST hits in the *P. yezoensis* genome when using plant and animal CBP-HATs as reference. CBP-HATs were also absent in the other unicellular and multicellular red algal genomes, indicating an ancient gene loss in the red algal ancestor.

Among the 10 *HDAC* genes, five were identified as encoding SIRT proteins. This gene number of SIRTs in *P. yezoensis* is higher than in some plants and green algae, e.g., two each in *Arabidopsis* and *Chlamydomonas* (Hollender and Liu, 2008; Chu and Chen, 2018). SIRTs usually harbor at least one sirtuin-type domain, which typically contains five alpha-helixes (Frye, 2000). Functional domain analysis revealed that each PySIRT harbored one sirtuin-type domain (**Figure 1B**). Interestingly, the sirtuin-type domain in Py06502 was split into two parts by 110 aa which was encoded by a 330 bp “spacer” sequence (**Figure S1**). Although the classic five alpha-helixes of the sirtuin domain and the Zn-binding site were observed in the predicted spatial conformation of Py06502, further experiments are still required to examine the effect of the “spacer”-encoding peptide on its enzymatic activities. Besides, in the duplicated paralog of Py06502 in *P. yezoensis*, Py07153, the “spacer” was of 97% identity in the nucleotide sequence. However, the reading frame in Py07153 was shifted by an extra intron starting from +43, leading to a false-translated N-terminal part of sirtuin-domain. Then the “spacer” sequence “corrected” the reading frame, resulting in a partial sirtuin-domain at the C-terminal. Additionally, the split domain was also observed in its Bangiales orthologs, such as *P. haitanensis*, *Porphyra umbilicalis* and *Bangia fuscopurpurea*, but not in other red algal species such as *Chondrus crispus* and *Porphyridium purpureum*, and neither in plants nor animals (**Figure S1**). The “spacer” sequences in Bangiales genomes were different in length, ranging from 312–750 bp, and exhibited only 46%-65% of sequence identity, much lower than the bilateral “sirtuin” region (53%-78%). Conservation of “split sirtuin domain” in Bangiales indicated that the “spacer” sequence was inserted in the ancestor of Bangiales, and then went through a higher mutation rate than the domain region.

The other five HDAC genes were identified as the *P. yezoensis* representatives of RPD3/HDA1 superfamily and named as PyHDA1, as they all harbored the typical conserved histone deacetylase domain (**Figure 1B**). The length of this domain was around 300aa long except for the much shorter one in Py09197. Additionally, Py04721 also possesses two Zn-binding sites embedded in its deacetylase domain. As for the plant-specific HD2, we failed to find any homologs in *P. yezoensis* and the other red algae genomes, confirming its exclusive existence in the green lineage and an evolutionary origin after the divergence of the red and green lineages.

### 3.2 Genome synteny of *HATs* and *HDACs* between *P. yezoensis* and *P. haitanensis*

**Figure 2:**
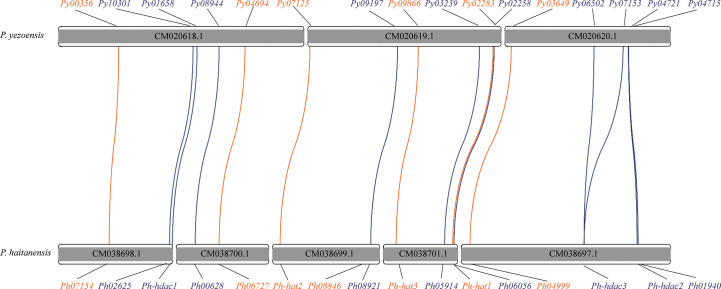
Synteny of HAT and HDAC genes in *P. yezoensis* genome and *P. haitanensis* genome. HAT genes are marked in brown and HDAC genes in blue. Contigs without the HAT and HDAC genes are not shown in the figure.

*HATs* and *HDACs* were distributed on the three chromosomes of *P. yezoensis*, e.g., two *HAT*s and three *HDAC*s on CM020618.1, three on CM020619.1 for each, as well as the last *HAT* and four *HDAC*s on CM020620.1 (**Figure 2**). Most *P. yezeoensis HAT*s and *HDAC*s had individual orthologous loci in the *P. haitanensis* genome (protein sequence similarity in each orthologous pair ranged from 85%-96%), showing strong syntenic relationships.

We noticed that two *P. haitanensis* loci were linked to *PyGCN5*-*Py03649* in the syntenic map. We doublechecked its orthologous relationships between *P. yezoensis* and *P. haitanensis*. When blasting against the *P. haitanensis* genome, the top two hits of *PyGCN5*-*Py03649* were *Ph04999* and *Ph08846*, with protein identity at 97% and 82%, respectively. Conversely, both *Ph04999* and *Ph08846*’s best hits against the *P. yezoensis* genome were *PyGCN5*-*Py03649*. However, the two *P. haitanensis* genes were located in different contigs, and moreover, were about 85% identical to each other, even less than the identity between *Py03649* and *Ph04999* (**Figure 2**; **Figure S2**). Therefore, we excluded the possibility of a tandem duplication and translocation event afterwards during the independent evolution of *P. haitanensis*. The plausible explanation is that Py03649 and Ph04999, which had higher protein sequence identity, were authentic orthologous pairs, and the *P. yezoensis* ortholog of *Ph08846* was lost during evolution. Different situations were observed for the two *HDAC*s, *PySIRT3-Py06502* and *PySIRT4-Py07153*. Both had the best hits in the *P. haitanensis* genome as *Ph-hdac3*. Although located at a large distance of 5000 Kb on CM020620.1 (**Figure 2**), the two genes shared higher identity (92%) in the translated protein sequence than either of them comparing to Ph-hdac3 (62% and 72% respectively) (**Figure S2**). Therefore, *PySIRT3-Py06502* and *PySIRT4-Py07153* are more likely to have originated from a duplication event during the independent evolution of *P. yezoensis*. The other two *HDAC* genes, *PyHDAC5-Py04721* and *PyHDAC3-Py04715*, located adjacently to each other with a 45 Kb interval, so did their orthologs in *P. haitanensis* (**Figure 2**). However, the sequence identity between proteins encoded by *PyHDAC5- Py04721* and *PyHDAC3-Py04715* was only 30%, also excluding the possibility of originating from a tandem duplication event.

### 3.3 Phylogenetic analysis of red algal HATs and HDACs

To elucidate the evolutionary history of HATs and HDACs in red algae, we identified their homologs in several red alga genomes including multicellular *Porphyra umbilicalis* and *Chondrus crispus*, as well as unicellular *Porphyridium purpureum* (Bhattacharya et al., 2013; Collen et al., 2013; Brawley et al., 2017). Then we generated the phylogenetic trees for each family. Well-studied homologs in the model organisms *Arabidopsis* and *Saccharomyces* were included in phylogeny analysis to assign the subfamily for red algal genes in an evolutionary cluster. *P. umbilicalis* also belongs to the Bangiales as *P. yezoensis* and *P. haitanensis* do. However, only 9 genes encoding two GCN5s, one GNAT, one MYST, two HDA1s, and three SIRTs, respectively, were found in its genome (**Table S2**). Considering the close evolutionary relationship to *P. yezoensis* and *P. haitanensis*, and its relatively fragmented genomic assembly, the lack of genes encoding certain subfamilies of HATs and HDACs in the current *P. umbilicalis* genome information might have mainly resulted from incomplete genome sequencing. In *Chondrus* and *Porphyridium*, gene members, with total numbers comparable to *P. yezoensis*, were identified for most of the subfamilies except the HAT1 and class IV HDA1. Further experimental work to amplify the adjacent genome fragments must determine whether the two subfamilies were actually absent in *Chondrus* due to gene loss events during evolution, or just mistakenly lost in genome sequencing and assembly.

**Figure 3:**
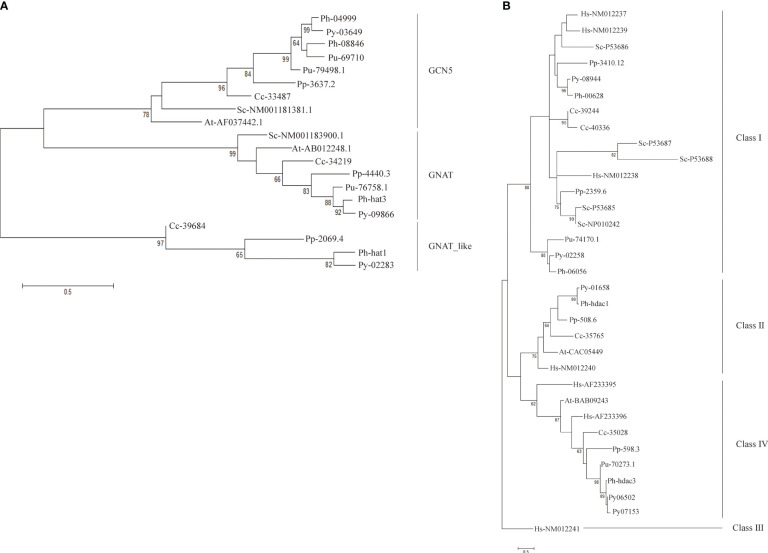
Phylogenetic trees of GNAT family **(A)** and SIRT family **(B)**. Each protein was named as the initial letters of the genera and species names, linked with the accession number or ID by a hyphen. Confidence levels for the tree branches that are best supported by bootstrap analysis are shown as numbers. Abbreviations for species are: Arabidopsis thaliana (At), Homo sapiens (Hs), Saccharomyces cerevisiae (Sc), Pyropia yezoensis (Py), Pyropia haitanensis (Ph), Porphyra umbilicalis (Pu), Chondrus crispus (Cc), Porphyridium purpureum (Pp).

Three clusters were clearly formed in the phylogenetic tree of GNAT-HAT family (**Figure 3A**). According to the included *Arabidopsis* homologs and conserved domains, they were annotated as GCN5, GNAT, and HAT-like respectively. In the GCN5 subfamily, *Py03649* and *Ph04999* were grouped together, neighboring with a sister cluster of *Ph08846* and a *P. umbilicalis* gene *Pu69710*, then another *P. umbilicalis* gene. The close phylogenic relationship of *Py03649* and *Ph04999* is consistent with our previous protein sequence analysis, further confirming our postulation of two paralogs existing in their Bangiales ancestor and then a specific gene loss event happening in the independent evolution of *P. yezoensis*. Considering that there is only one homolog for *Porphyridium* and *Chondrus*, it is logical to further deduce that the two GCN5 paralogs originated from a gene duplication event after the divergence of Bangiales. Additionally, *PyGNAT-like-Py02283* located along with a few of its red algal homologs, without the presence of any plant homologs, suggesting that they diverged significantly from the other GNAT clusters in red algae. *PyGNAT*-*Py09866*, like MYST and TAFII250 genes, located separately on the phylogenetic tree along with its sole homolog from other red algal species, exhibiting a high conservation in red algae (**Figures S3A, B**).

For the HDA1 family, the five *P. yezoensis* genes were grouped into three clusters named Class I, Class II, and Class IV (**Figure S3C**). The two Class I genes, *Py10301* and *Py03239*, formed two separate clusters with their red algal homologs, respectively, suggesting that they diverged long ago in their red algal common ancestor. Eukaryotic Sir2-like proteins group into four main branches as classes I–IV (Frye, 2000; Pandey et al., 2002). *Py08944* and its red algal homologs were clustered with human and yeast Class I SIRT proteins, thus designated as Class I SIRTs. *Py02258* and its Bangiales homologs formed a sister cluster of Class I SIRTs, indicating that they may be a Bangiales-specific Class I group divergent from the classic one. The phylogenetic analysis then designated *Py01658* and *Py06502* (as well as its duplicate *Py07153*) to be Class II and Class IV SIRTs respectively. As in *Arabidopsis*, Class III SIRT was not found in the red algal species (**Figure 3B**) (Pandey et al., 2002).

**Figure 4:**
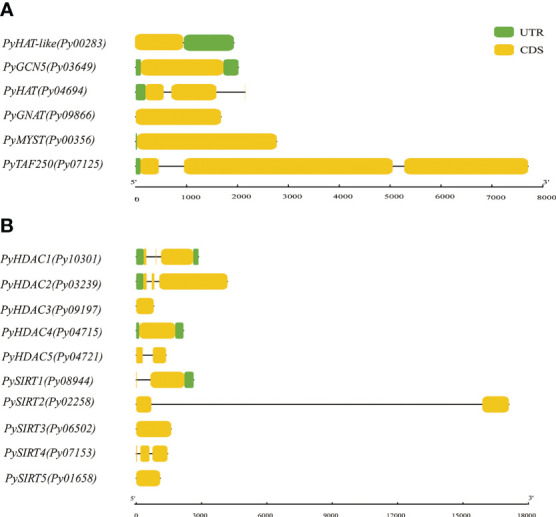
Gene structure of P. yezoensis HAT **(A)** and HDAC **(B)** genes. CDS regions in exon(s) and intron(s) are shown in yellow boxes and black lines, respectively. The UTR regions (5/& 3/) are represented by green boxes.

To obtain more insights into gene evolution, the exon/intron structures of the *PyHAT* and *PyHDAC* genes were investigated. Of the six *PyHAT* genes, four have no introns and the other two have one and two introns, respectively. For the *PyHDAC* genes, four have one intron and two have two introns (**Figure 4**). Overall, the average intron number is 0.688 per gene, comparable to the value in genome-wide (0.60) (Wang et al., 2020), but much less than the value of their plant counterparts [ranging from 0 to 78 in *Arabidopis* (Wang et al., 2020)]. Besides, a similar gene structure was observed in gene members from the same subgroup in plant *HAT*s and *HDAC*s (Pandey et al., 2002, Liu et al., 2012; Du et al., 2022). Accordingly, *Py10301* and *Py03239*, being the closet members from the Class I-*HDA* subfamily, both had two introns, despite of a longer 3^rd^ exon in *Py03239*. However, inconsistency in the exon/intron organization was observed for the two Class IV-*HDA1* genes *Py04721* and *Py09197*, reflecting the high diversity of gene structure established in the evolutionary history of *Pyropia* species.

### 3.4 Subcellular localization

**Figure 5:**
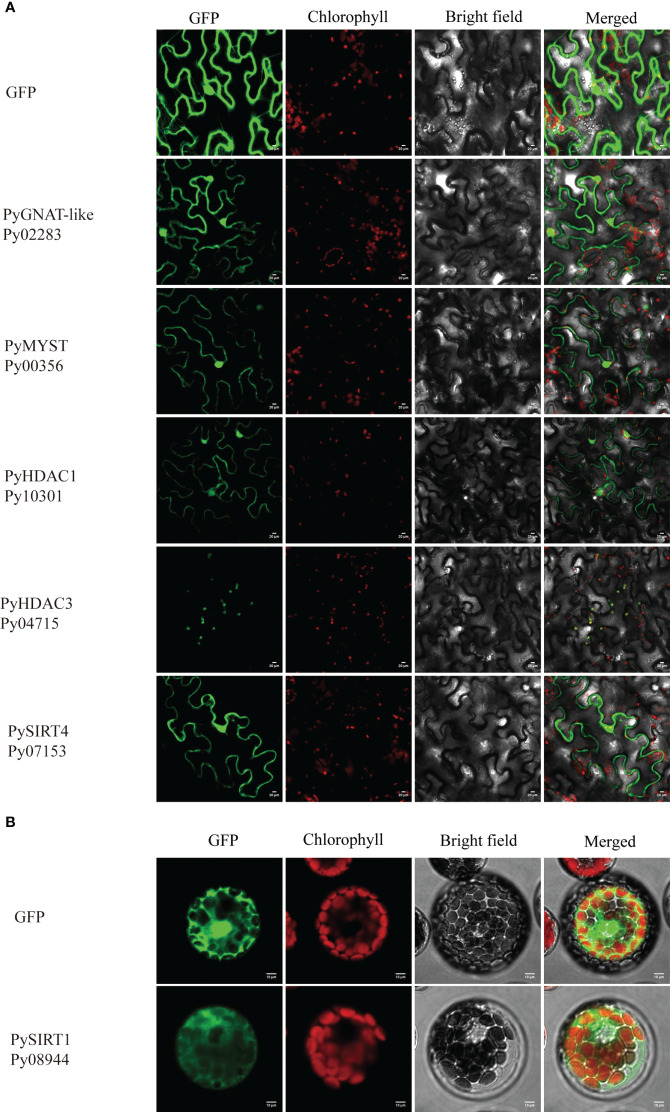
Subcellular localization of HAT and HDAC genes in tobacco leaves **(A)** and *Arabidopsis* protoplasts **(B)**. The fluorescence channels of the target protein, chloroplast, bright field and superposition are shown from left to right, respectively. Scale bars=10 μm. 35S:GFP was used as a negative control.

We applied three different subcellular localization prediction programs to determine the likely subcellular localization of HATs and HDAC proteins. Unfortunately, these programs gave different results for each protein. For example, Plant-mPLoc predicted all of them to be in the nucleus, except PySIRT2-Py02258, which was predicted to be in chloroplast. Nuclear localization signals were also detected in eight of these proteins. However, PSORT generated the same nucleus localization for only six of them and WoLF PSORT only found for three proteins the nuclear localization with high confidence (**Table S3**). Therefore, the computational prediction failed to provide convincing subcellular localizations. Because GFP-transgenic technology is not available for *P. yezoensis* yet, we next investigated the subcellular localization of several PyHATs and PyHDACs using transient expression of green fluorescent protein (GFP) fusion proteins in tobacco leaves and *A. thaliana* protoplasts. However, transcriptions of *PyHAT-like-Py04694*, *PyHDAC4*-*Py09197*, *PyHDAC5-Py04721* and *PySIRT2-Py02258* were not detected using either thalli or conchocelis to prepare cDNA templates. Their *P. haitanensis* orthologs were also transcriptionally undetectable using corresponding cDNA templates. This is consistent with the low FPKM values revealed in previous transcriptomic data of the two heteromorphic generations (Wang et al., 2020). These genes were not analyzed further. Besides, we failed to amplify the full-length cDNA of *Py03649*, *Py08966*, *Py04694*, *Py07125*, *Py03239*, *Py09197*, *Py04721*, *Py02258*, *Py06502* and *Py01658*, thus, only the following six genes were subject to transient expression analysis. Fluorescence signals of GFP-fused PyGNAT-like-Py02283, PyMYST-Py00356, PyHDAC1-Py10301, and PySIRT4-Py07153 were detected in the nucleus and cytosol of tobacco cells, suggesting potential roles of the three genes the respective compartments (**Figure 5A**). The GFP signal of PyHDAC3-Py04715 overlapped well with auto-fluorescence of chlorophyll, suggesting its chloroplast localization and potential role in the post-translational modification of chloroplast proteins. Since GFP-PySIRT1-Py08944 cassette was not successfully expressed in tobacco, we transformed it into an *Arabidopsis* protoplast and observed its expression in the cytosol (**Figure 5B**).

### 3.5 Expressional levels under dehydration

**Figure 6:**
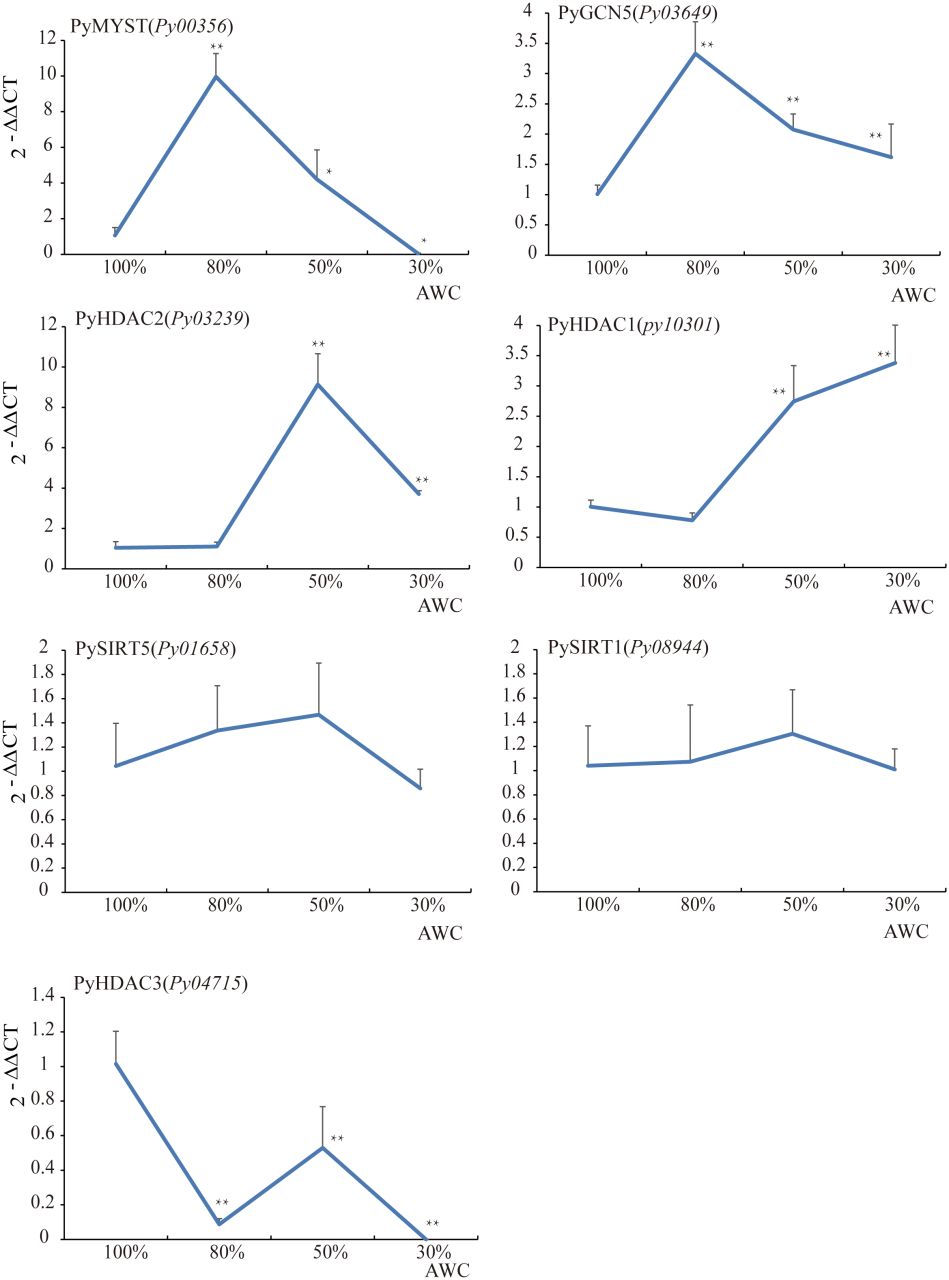
The relative expression analysis of HAT and HDAC genes under dehydration in *P. yezoensis*. The X-axis represents different degrees of water content (AWC) and the Y-axis represents the relative expression of specific HAT and HDAC gene. Data represent the mean value ± standard deviation (SD) (n = 3). Asterisks represent significant differences at P ≤ 0.05 (*) and P ≤ 0.01 (**) when comparing to AWC100.

To gain insight into the potential role of *HAT* and *HDAC* genes in the stress response, we next investigated the differential transcription of seven *HATs* and *HDAC*s under different degrees of water loss *via* qRT–PCR (**Table S1**, **Figure 6**). Transcription of *PyMYST-Py00356* and *PyGCN5-Py03649*, significantly increased to 10.0 and 3.3 folds, respectively, under 30% of water loss relative to that before dehydration, followed by down-regulation under higher dehydration. The transcriptional level of *PyMYST-Py00356* even dropped to much less than the value under normal conditions. GCN-5 was reported to be a master regulator of responses to environmental stimuli in plants (Kim et al., 2020). MYST in soybean is dominantly expressed in roots and is highly responsive to abiotic stresses (Feng et al., 2022). The transient up-regulation of the two *HAT* genes hand in hand with their nuclear localization, indicated the important role they played in the early response to dehydration. The transcription of two class I-*HDA1* genes, *PyHDAC1-Py10301* and *PyHDAC2-Py03239*, barely changed under early dehydration, but elevated to more than 9.1 and 2.7 folds upon 50% of water loss relative to that before dehydration, as shown in **Figure 6**. Different from the subsequent down-regulation of *PyHDAC2-Py03239* at 70% of water loss, *PyHDAC1-Py10301*, which encoded a nuclear-localized protein, exhibited progressive up-regulation and reached 3.4 folds of the value under normal conditions. The transcription of the other two *SIRT-HDAC* genes, *Py01658* and *Py08944*, hardly varied along the full course of dehydration, with foldchanges less than 2. For *PyHDAC3-Py04715*, we observed a great reduction in transcription at 30% of water loss, though followed by slight up-regulation to half of the level before dehydration and down-regulated to an even lower level at 70% of water loss than in the earlier stage (**Figure 6**). Considering the cytosol and chloroplast localization of Py*SIRT1-Py08944* and *PyHDAC3-Py04715*, respectively, they may not make important contributions in regulating down-stream gene expression in response to water loss.

### 3.6 Inhibiting HDAC activity accelerated the reduction in photosynthesis efficiency during dehydration

**Figure 7:**
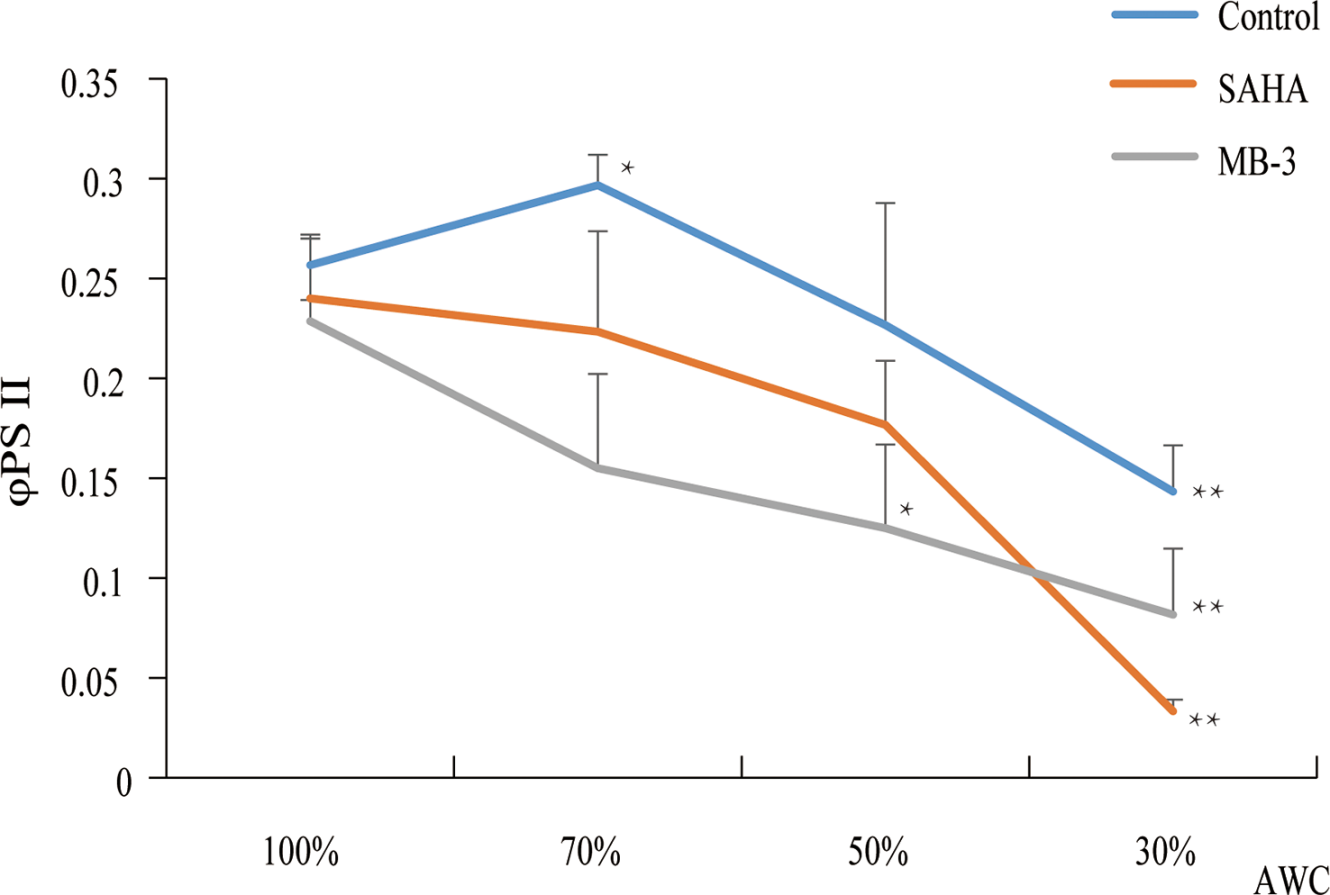
Photosynthetic parameters (ΦPSII) changes of *P. yezoensis* thalli under dehydration with SAHA and MB-3 treatments. The Y-axis indicates the value of φPSII and the X-axis represents different absolute water contents (AWC) of dehydrated samples. Data are the means of three independent experiments ( ± SD). Asterisks represent significant differences at P ≤ 0.05 (*) and P ≤ 0.01 (**) when comparing to AWC100.

In plants, the reduction in the enzymatic activities of HAT or HDACs in gene mutants or *via* specific chemical inhibitors repressed the tolerance to abiotic or biotic stresses (Hu et al., 2015). Since we found that the expression of *HAT* and *HDAC* genes was induced by dehydration in *P. yezoensis*, to further investigate the epigenetic regulation by histone acetylation in response to dehydration stress, we treated *P. yezoensis* thalli with SAHA (specific HDAC inhibitor) and MB-3 (specific HAT inhibitor) respectively for 24 h and then exposed them to air for dehydration. Photosynthesis efficiency was detected before dehydration and after 30%, 50% and 70% water loss (**Figure 7**). Before dehydration, there were no significant differences in ΦPSII value between control and treated thalli. Control thalli exhibited a slight increase in ΦPSII value upon 30% of water loss, then decreased significantly upon more intense desiccation and declined to less than 0.15 upon 70% of water loss. However, ΦPSII value of SAHA-treated thalli presented an immediate decline at the early stage of dehydration and went down to nearly zero upon 70% of water loss, suggesting the photosynthesis activity was greatly repressed by SAHA-treatment. An even sharper decline in ΦPSII value at an early stage was observed in MB-3-treated thalli. Apparently, either inhibiting HAT by MB-3 or HDAC by SAHA negatively affected the dehydration tolerance of *P. yezoensis* thalli. Combining the induced expression of both *HAT* and *HDAC* genes by dehydration, this suggests their essential roles in generating enormous variations in the genomic pattern of histone acetylation upon dehydration, thereby triggering transcriptional changes of downstream genes to adjust physiological and metabolic activities for stress acclimation.

### 3.7 The effect of MB-3 and SAHA on transcriptional patterns of dehydration-responsive genes

**Figure 8:**
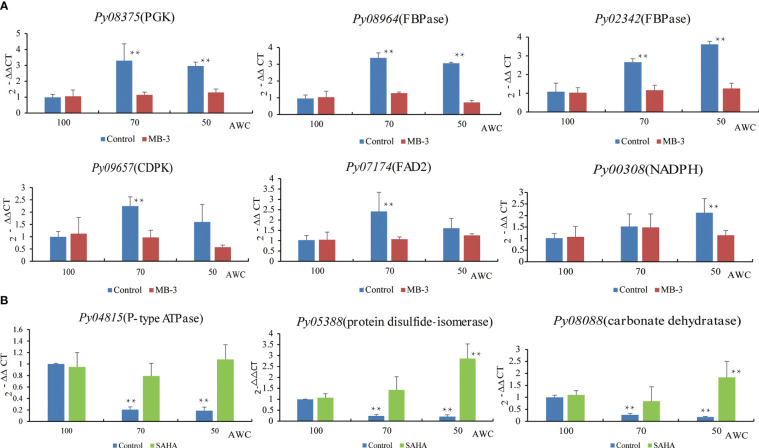
Expression analysis of dehydration-responsive genes under MB-3 treatment **(A)** and SAHA treatment **(B)** in *P. yezoensis*. The X-axis represents different degrees of water content (AWC) and the Y-axis represents the relative expression of specific HAT and HDAC gene. Different treatments were indicated by different colors. Data represent the mean value ± standard deviation (SD) (n = 3). Asterisks represent significant differences at P ≤ 0.05 (*) and P ≤ 0.01 (**) when comparing to AWC100.

Many genes were previously reported to be responsive to osmotic stress in plants, such as the fructose-1,6-biphosphatase (FBPase) and phosphoglycerate kinase (PGK) related to sucrose production (Daie, 1993), delta-12 fatty acid desaturase 2 (FAD2) for promoting the production of unsaturated fatty acids (Dar et al., 2017), the Ca^2+^ sensor calcium-dependent protein kinases (CDPKs) to trigger calcium signaling(Gao et al., 2014), as well as NADPH oxidase for cellular redox balance (Ju et al., 2020). We first analyzed the expression patterns of their *Pyropia* homologs at 70% and 50% AWC (**Figure 8**). Two FBPase genes and one PGK gene in *P. yezoensis* displayed strong up-regulation at both 70% and 50% AWC. Transcriptional level of delta-12 fatty acid desaturases FAD2 (*Py07174*) increased by more than two folds at 70% AWC, albeit dropped to a level slightly higher than that one at 100% AWC. The transcriptional pattern of the calcium-dependent protein kinase CDPK (*Py09657*) was similar to FAD2. A gene encoding the NADPH oxidase 5 exhibited gradual increase in transcription from 1.5 folds at 70% AWC to 2.2 folds at 50% AWC. To determine whether these dehydration-responsive genes were regulated by histone acetylation, we then investigated the variations in transcriptional patterns in response to dehydration under MB-3 or SAHA treatment. We observed that except for a slight increase in NADPH oxidase 5 at 70% AWC and a down-regulation of CDPK at 50% AWC, transcriptional levels of all the six genes barely changed after water loss when treated with MB-3. However, when treated with SAHA, the up-regulation observed in the control medium after water loss was also depressed, though at variable degrees. We further searched out three genes that were previously reported to be significantly down-regulated under dehydration stress *via* transcriptomic analysis, including P-type ATPase, carbonic anhydrase and protein disulfide-isomerase (Wang et al., 2020). A substantial reduction in transcription at both 70% and 50% AWC in normal medium was confirmed by RT-qPCR analysis. By contrast, when treated with SAHA, transcription of P-type ATPase was unchanged.

## 4 Conclusion

Recent studies show that a wealth of genes exhibit transcriptionally changes in response to abiotic stresses, particularly osmotic stress resulting from salt treatment or dehydration in *P. yezoensis*. Histone acetylation and deacetylation play essential roles in transcriptional regulation in response to stress in plants, albeit little information is available on genes responsible for these processes in *P. yezoensis* and in general red algal species. In this study, we identified 6 *HAT* genes and 10 *HDAC* genes in *P. yezoensis* and their corresponding homologs in several other red algal genomes. Putative gene loss and gene duplication events were deduced in the *P. yezoensis* genome through chromosome location, protein sequence analysis and phylogenetic analysis. *HAT* and *HDAC* genes were assigned to the corresponding subfamilies according to the conserved domains they encoded. CBP-HAT and plant-specific HD2-HDAC were absent in all red algal lineages. Subcellular localization and transcriptional analysis as well as inhibitory experiments of HAT and HDAC revealed that histone acetylation and deacetylation make important contributions in response to dehydration stress in *P. yezoensis.* Moreover, *PyGCN5*, *PyMYST* and *PyHDA1* genes, which exhibited increased expressional levels under dehydration, were the main players. Our results lay a solid foundation for research regarding histone modification-mediated stress response regulation in nori and red algae. We also notice that intensive experimental studies are required to further validity the biological functions of these genes and identify the target genes that are regulated by remodeling histone acetylation/deacetylation. This study provides essential information for understanding the regulatory network in stress adaptation in red algae.

## Data availability statement

The original contributions presented in the study are included in the article/**Supplementary Material**. Further inquiries can be directed to the corresponding author. Correspondence and requests for materials should be addressed to DW (wangdm@ouc.edu.cn).

## Author contributions

DW designed the research. ZZ and HQ performed research. ZW, YP, and XG prepared samples. DW and AP contributed in funding and paper editing. ZZ and DW analyzed data and wrote the paper. All authors contributed to the article and approved the submitted version.
