# Supplementary figures and images for: Characterization of histone acetyltransferases and deacetylases and their roles in response to dehydration stress in Pyropia yezoensis (Rhodophyta)
Source: Front Plant Sci. 2023 May 16;14:1133021. doi: 10.3389/fpls.2023.1133021 (PMC10227436; doi:10.3389/fpls.2023.1133021)

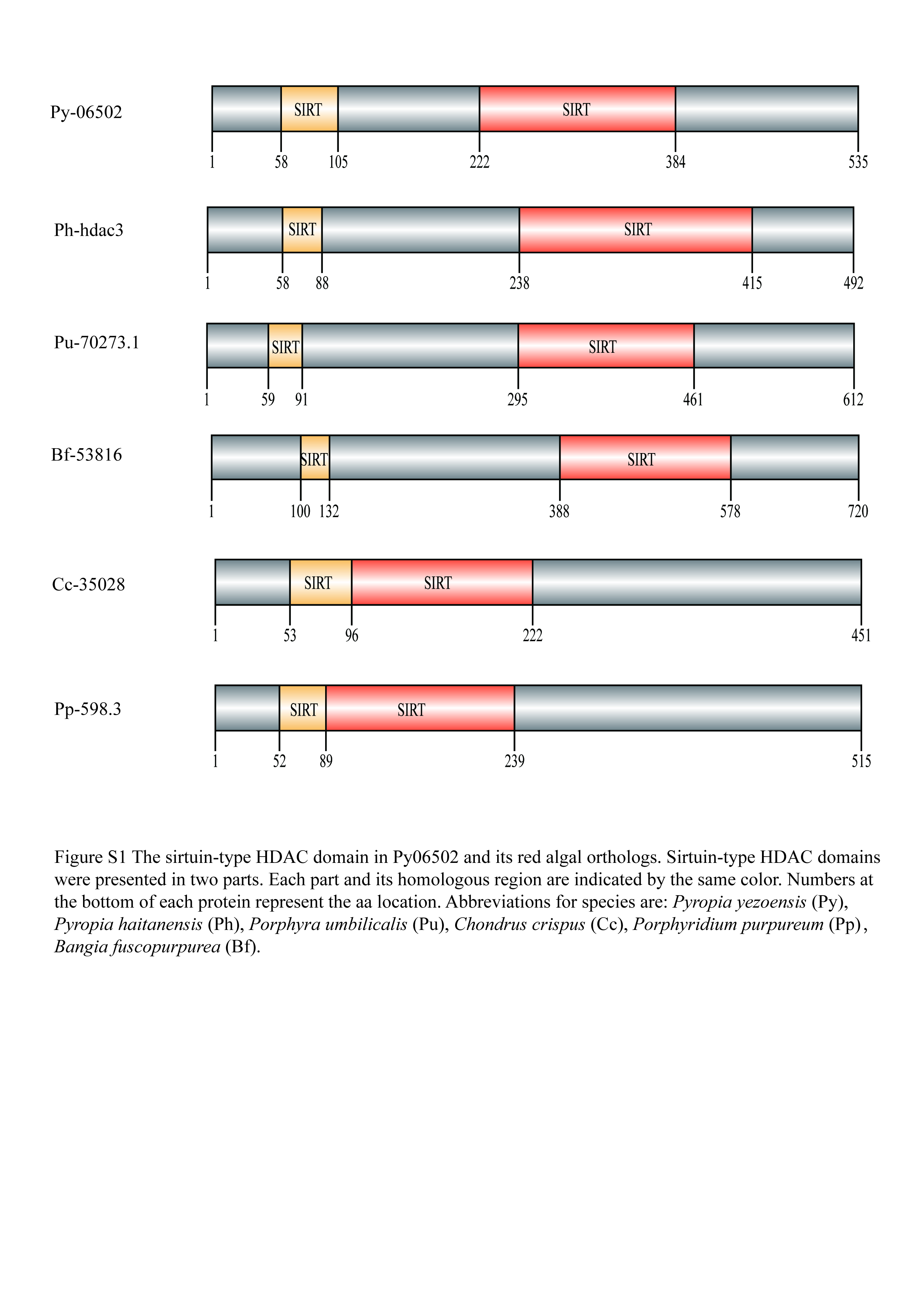

Supplement: Supplementary file 1 [file Image_1.tif]

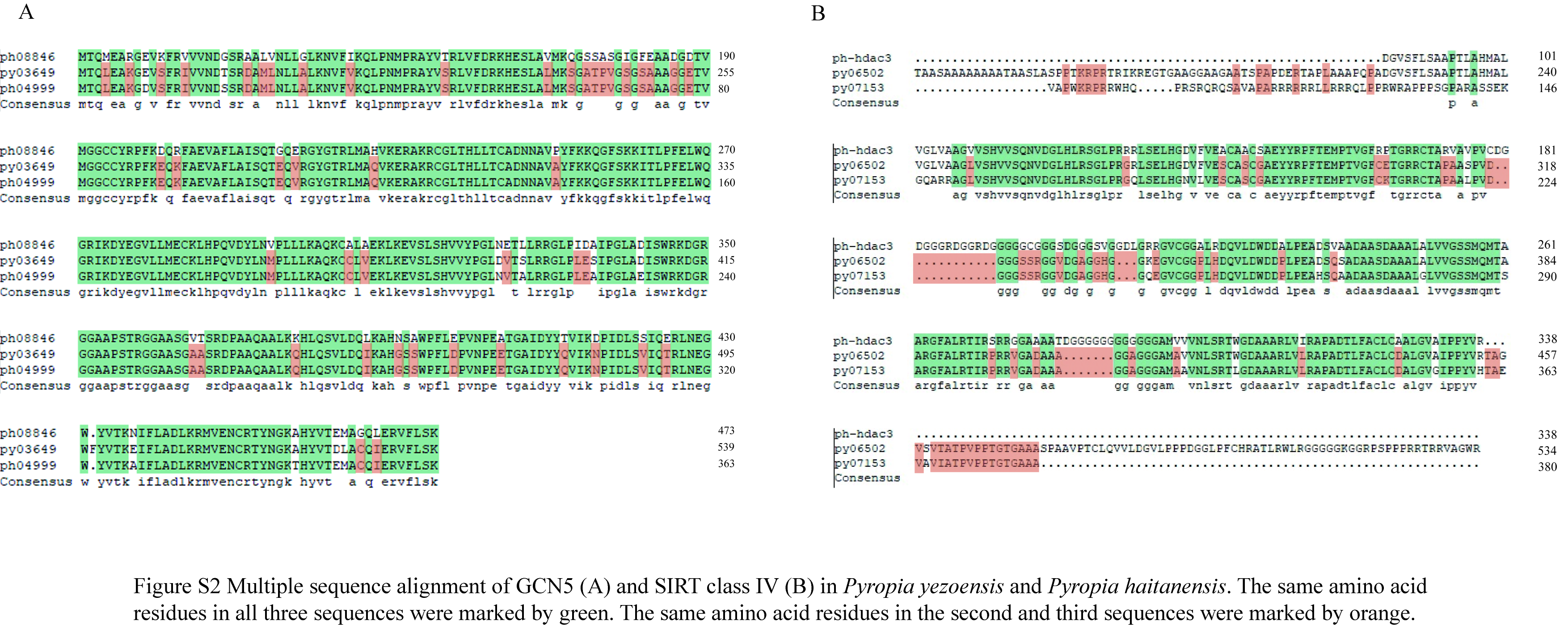

Supplement: Supplementary file 2 [file Image_2.tif]

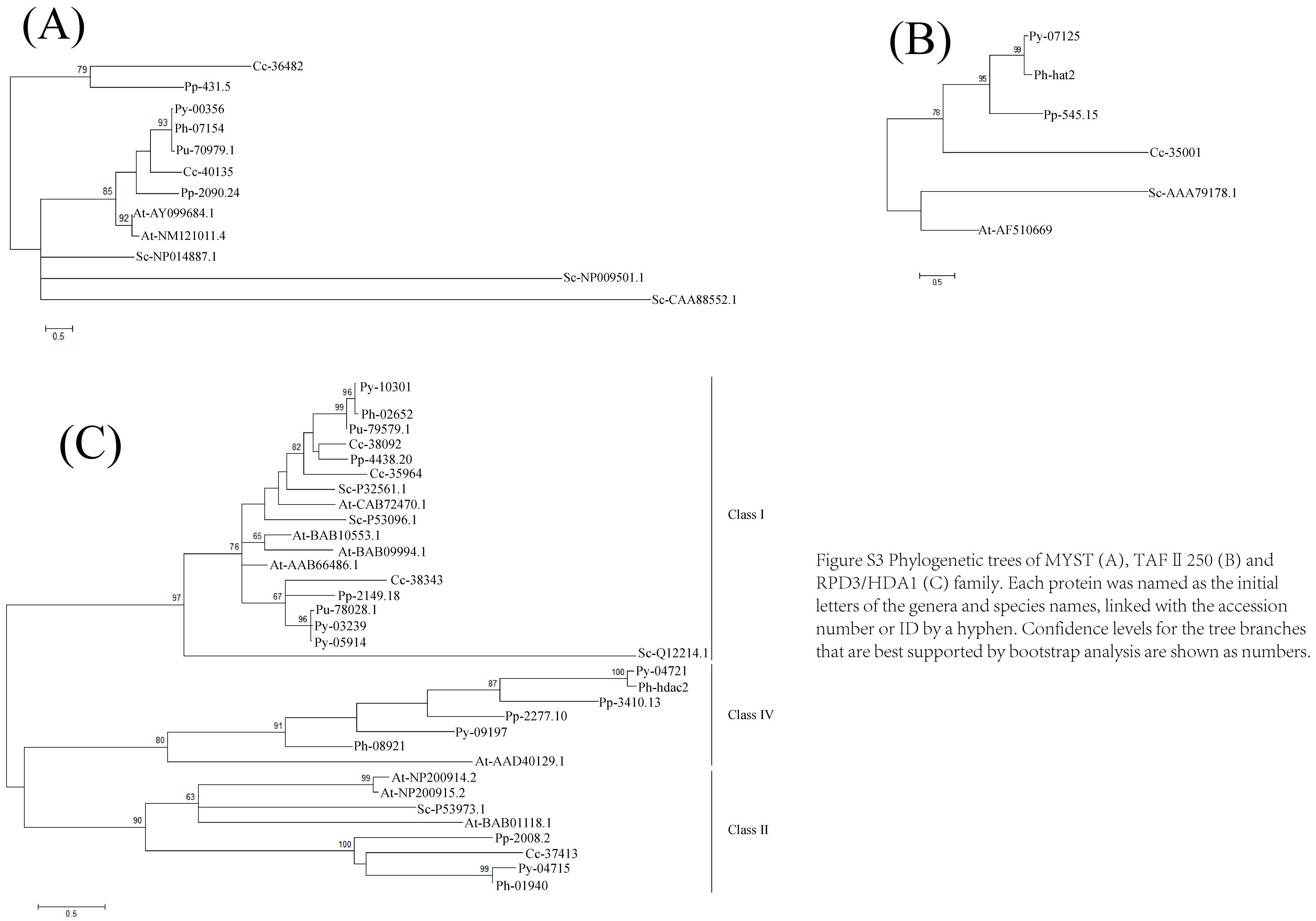

Supplement: Supplementary file 3 [file Image_3.tif]
