# Supplementary material for: Characterization of histone acetyltransferases and deacetylases and their roles in response to dehydration stress in Pyropia yezoensis (Rhodophyta)
Source: Front Plant Sci. 2023 May 16;14:1133021. doi: 10.3389/fpls.2023.1133021 (PMC10227436; doi:10.3389/fpls.2023.1133021)
Supplement: Supplementary file 4 [file Table_1.docx]

Table S1

(a)Sequence information of primers used in constructing plant expression cassettes

| Primer Name | Sequence Information (5’ –3’) |
| --- | --- |
| *Py02283* | F ATGGCGTCGTGCTACACTG  R TACCAGGCTCCGGTACAGGA |
| *Py00356* | F ATGCCTGGCCGCTCCAACT  R GCGTCGCCTTCCCGCAG |
| *Py10301* | F ATGTCGCGTCCGCGTGTG  R CGGAACCGAGGGGGTGGG |
| *Py04715* | F ATGGGCCCCTCCCCTTTGAC  R GCCCCCCCCAGGTGGG |
| *Py07153*  *Py08944* | F ATGCCGACGGTGGGCTT  R GGCGGCCGCCCCAGTC  F ATGACGCCGCCGTCG  R CATGACCGCCTCCGGC |

(b)Sequence information of primers used in qPCR

| Primer Name | Sequence Information (5’ –3’) |  |
| --- | --- | --- |
| *Py00356* | F ACACCAAGTCCTCCAGCCC |  |
|  | R ACACCAAGTCCTCCAGCCC |  |
| *Py03649* | F CAAGGGCGAGGTTTCATT |  |
|  | R AAAAACGAGGCGGGATAC |  |
| *Py03239* | F GCAACTTCTACTATGCGCAGG |  |
|  | R TACTCATCGGCGTGGAACC |  |
| *Py10301* | F TCGCACCGTTACCCCTGA  R GCCTCCCGCCCAGTTGAT |  |
| *Py01658* | F GCGGTGGGGACGTTTGTC |  |
|  | R ACGCCGAGTAGACGGTGAAG |  |
| *Py08944* | F GGCGGCTCGTCCTCCTCA |  |
|  | R GACCCCGCCCCGTTTTA |  |
| *Py04715* | F GATTCTGGATTGGGATGTGC |  |
| *Py08375*  *Py08964*  *Py02342*  *Py09657*  *Py07174*  *Py00308*  *Py04815*  *Py05388*  *Py08088* | R GTGCCACATTGAGGATGTTG  F CAAGGTGCTGCTGTCTTCT  R GGCCATCTTCACATCCTTCTT  F ATGGAGGAGTCGCCTATGA  R ATCGAAGCCATCAGGTTCTG  F GCAACGACCGAGTACATCAA  R GTAGATGAAGACACCGCCATAC  F TTTGGCCTATCCAACATCCTC  R ACAAGTCCACCTTCTCATCATAC  F CTCAGTGAACAATGCGTTTGG  R TCCATGTGGTTGGTGTTCTT  F TGGCGTTTACGTGCTCAT  R ATCGCTGCCAGACATCTTC  F GTGACGACCTTTGCCATCT  R AGAGCAAAGAGGAGGGAGAA  F GCCAACAAAGGTAACGACAAG  R GTCCGATGAGAACGAGTAGATG  F GCAGTCGCCCATTGACTT  R GCAATTGTACTTCACGCCATTT |  |
