# Supplementary material for: Characterization of histone acetyltransferases and deacetylases and their roles in response to dehydration stress in Pyropia yezoensis (Rhodophyta)
Source: Front Plant Sci. 2023 May 16;14:1133021. doi: 10.3389/fpls.2023.1133021 (PMC10227436; doi:10.3389/fpls.2023.1133021)
Supplement: Supplementary file 5 [file Table_2.docx]

Table S2

(A)HAT genes identified in red algal genomes, including *Pyropia haitanensis*, *Porphyra umbilicalis*, *Chondrus crispus* and *Porphyridium purpureum*.

| species | Family | Sub-family | Gene ID |
| --- | --- | --- | --- |
| *Chondrus crispus* | GNAT | GCN5 | *Cc33487* |
| *Pyropia haitanensis* |  |  | *Ph04999* |
| *Pyropia haitanensis* |  |  | *Ph08846* |
| *Porphyridium purpureum* |  |  | *Pp3637.2* |
| *Porphyra umbilicalis* |  |  | *Pu69710.1* |
| *Porphyra umbilicalis* |  |  | *Pu79498.1* |
|  |  |  |  |
| *Chondrus crispus* |  | HAT-like | *Cc39684* |
| *Porphyridium purpureum* |  |  | *Pp2069.4* |
| *Pyropia haitanensis* |  |  | *Ph-hat1* |
|  |  |  |  |
| *Chondrus crispus* |  | GNAT | *Cc34219* |
| *Pyropia haitanensis* |  |  | *Ph-hat3* |
| *Porphyridium purpureum* |  |  | *Pp4440.3* |
| *Porphyra umbilicalis* |  |  | *Pu76758.1* |
|  |  |  |  |
| *Pyropia haitanensis* | HAT1 |  | *Ph06727* |
| *Porphyridium purpureum* |  |  | *Pp3524.14* |
|  |  |  |  |
| *Porphyra umbilicalis* | MYST |  | *Pu70979.1* |
| *Pyropia haitanensis* |  |  | *Ph07154.t1* |
| *Chondrus crispus* |  |  | *Cc40135* |
| *Porphyridium purpureum* |  |  | *Pp2090.24* |
| *Porphyridium purpureum* |  |  | *Pp431.5* |
| *Chondrus crispus* |  |  | *Cc36482* |
|  |  |  |  |
| *Pyropia haitanensis* | TAFⅡ250 |  | *Ph-hat2* |
| *Porphyridium purpureum* |  |  | *Pp545.15* |
| *Chondrus crispus* |  |  | *Cc35001* |

(B)HDAC genes identified in red algal genomes, including *Pyropia haitanensis*, *Porphyra umbilicalis*, *Chondrus crispus* and *Porphyridium purpureum*.

| species | Family | Sub-family | Gene ID |
| --- | --- | --- | --- |
| *Chondrus crispus* | RPD3/HDA1 | ClassⅠ | *Cc38343* |
| *Chondrus crispus* |  |  | *Cc38092* |
| *Chondrus crispus* |  |  | *Cc35964* |
| *Pyropia haitanensis* |  |  | *Ph02652* |
| *Pyropia haitanensis* |  |  | *Ph05914* |
| *Porphyridium purpureum* |  |  | *Pp4438.20* |
| *Porphyridium purpureum* |  |  | *Pp2149.18* |
| *Porphyra umbilicalis* |  |  | *Pu79579.1* |
| *Porphyra umbilicalis* |  |  | *Pu78028.1* |
|  |  |  |  |
| *Chondrus crispus* |  | ClassⅡ | *Cc37413* |
| *Pyropia haitanensis* |  |  | *Ph01940* |
| *Porphyridium purpureum* |  |  | *Pp2008.2* |
|  |  |  |  |
| *Pyropia haitanensis* |  | ClassⅣ | *Ph08921* |
| *Pyropia haitanensis* |  |  | *Ph-hdac2* |
| *Porphyridium purpureum* |  |  | *Pp2277.10* |
| *Porphyridium purpureum* |  |  | *Pp3410.13* |
|  |  |  |  |
| *Chondrus crispus* | SIRT | ClassⅠ | *Cc39244* |
| *Chondrus crispus* |  |  | *Cc40336* |
| *Pyropia haitanensis* |  |  | *Ph06056* |
| *Pyropia haitanensis* |  |  | *Ph00628* |
| *Porphyridium purpureum* |  |  | *Pp2359.6* |
| *Porphyridium purpureum* |  |  | *Pp3410.12* |
| *Porphyra umbilicalis* |  |  | *Pu74170.1* |
|  |  |  |  |
| *Chondrus crispus* |  | ClassⅡ | *Cc35765* |
| *Pyropia haitanensis* |  |  | *Ph-hdac1* |
| *Porphyridium purpureum* |  |  | *Pp508.6* |
|  |  |  |  |
| *Chondrus crispus* |  | ClassⅣ | *Cc35028* |
| *Pyropia haitanensis* |  |  | *Ph-hdac3* |
| *Porphyridium purpureum* |  |  | *Pp598.3* |
| *Porphyra umbilicalis* |  |  | *Pu70273.1* |

(C)HAT genes identified in classic model organisms, including *Arabidopsis thaliana*, *Saccharomyces cerevisiae*.

| species | Family | Sub-family | Accession ID |
| --- | --- | --- | --- |
| *Arabidopsis thaliana* | GNAT | GCN5 | At-AF037442.1 |
| *Saccharomyces cerevisiae* |  |  | Sc-NM001181381.1 |
| *Arabidopsis thaliana* |  | GNAT | At-AB012248.1 |
| *Saccharomyces cerevisiae* |  |  | Sc-NM001183900.1 |
|  |  |  |  |
| *Arabidopsis thaliana* | HAT1 |  | At-BAB098892.1 |
| *Saccharomyces cerevisiae* |  |  | Sc-U33335.1 |
|  |  |  |  |
| *Arabidopsis thaliana* | MYST |  | At-AY099684.1 |
| *Arabidopsis thaliana* |  |  | At-NM121011.4 |
| *Saccharomyces cerevisiae* |  |  | Sc-NP014887.1 |
| *Saccharomyces cerevisiae* |  |  | Sc-NP009501.1 |
| *Saccharomyces cerevisiae* |  |  | Sc-CAA88552.1 |
|  |  |  |  |
| *Arabidopsis thaliana* | TAFⅡ250 |  | At-AF510669.1 |
| *Saccharomyces cerevisiae* |  |  | Sc-AAA79178.1 |

(D)HDAC genes identified in classic model organisms, including *Arabidopsis thaliana*, *Saccharomyces cerevisiae* and *Homo sapiens*.

| species | Family | Sub-family | Accession ID |
| --- | --- | --- | --- |
| *Arabidopsis thaliana* | RPD3/HDA1 | ClassⅠ | At-CAB72470.1 |
| *Arabidopsis thaliana* |  |  | At-BAB10553.1 |
| *Arabidopsis thaliana* |  |  | At-BAB09994.1 |
| *Arabidopsis thaliana* |  |  | At-BAB66486.1 |
| *Saccharomyces cerevisiae* |  |  | Sc-P32561.1 |
| *Saccharomyces cerevisiae* |  |  | Sc-P53096.1 |
| *Saccharomyces cerevisiae* |  |  | Sc-Q12214.1 |
|  |  |  |  |
| *Arabidopsis thaliana* |  | ClassⅡ | At-NP200914.2 |
| *Arabidopsis thaliana* |  |  | At-NP200915.2 |
| *Arabidopsis thaliana* |  |  | At-BAB01118.1 |
| *Saccharomyces cerevisiae* |  |  | Sc-P53973.1 |
|  |  |  |  |
| *Arabidopsis thaliana* |  | ClassⅣ | At-AAD40129.1 |
|  |  |  |  |
| *Saccharomyces cerevisiae* | SIRT | Class Ⅰ | Sc-P53685 |
| *Saccharomyces cerevisiae* |  |  | Sc-P53686 |
| *Saccharomyces cerevisiae* |  |  | Sc-P53687 |
| *Saccharomyces cerevisiae* |  |  | Sc-P53688 |
| *Saccharomyces cerevisiae* |  |  | Sc-NP010242 |
| *Homo sapiens* |  |  | Hs-NM012238 |
| *Homo sapiens* |  |  | Hs-NM012237 |
| *Homo sapiens* |  |  | Hs-NM012239 |
|  |  |  |  |
| *Arabidopsis thaliana* |  | Class Ⅱ | At-CAC05449 |
| *Homo sapiens* |  |  | Hs-NM012240 |
|  |  |  |  |
| *Homo sapiens* |  | Class Ⅲ | Hs-NM-012241 |
|  |  |  |  |
| *Arabidopsis thaliana* |  | Class Ⅳ | At-BAB09243 |
| *Homo sapiens* |  |  | Hs-AF233396 |
| *Homo sapiens* |  |  | Hs-AF233395 |
