# Supplementary material for: Characterization of histone acetyltransferases and deacetylases and their roles in response to dehydration stress in Pyropia yezoensis (Rhodophyta)
Source: Front Plant Sci. 2023 May 16;14:1133021. doi: 10.3389/fpls.2023.1133021 (PMC10227436; doi:10.3389/fpls.2023.1133021)
Supplement: Supplementary file 6 [file Table_3.docx]

Table S3

The subcellular localization of HATs and HDACs computationally predicted by different software.

| PyHDAC | Plant-mPLoc | PSORT Prediction (plant model) | predalgo | Wolf PSOR (plant) |
| --- | --- | --- | --- | --- |
| Py10301 | Golgi apparatus. Nucleus. | mitochondrial matrix\ lysosome \nucleus | O | cyto: 6, chlo: 4, mito: 4 |
| Py03239 | Nucleus. | microbody (peroxisome) |  | nucl: 5, chlo: 4, cyto: 2, mito: 2 |
| Py09197 | Nucleus. | microbody (peroxisome)\nucleus |  | chlo: 7, cyto: 4, mito: 3 |
| Py04715 | Chloroplast. Nucleus. | cytoplasm |  | chlo: 14 |
| Py04721 | Chloroplast. Nucleus. | mitochondrial intermembrane |  | chlo: 12, mito: 2 |
| Py08944 | Nucleus. | plasma membrane | C | chlo: 8, plas: 2, extr: 2, vacu: 1 |
| Py02258 | Chloroplast. | mitochondrial inner membrane\ plasma membrane | | plas: 8, chlo: 3, E.R.: 3 |
| Py06502 | Chloroplast. Nucleus. | mitochondrial inner membrane |  | chlo: 4, plas: 3, nucl: 2, cyto: 2, E.R.: 2 |
| Py07153 | Cytoplasm. Nucleus. | mitochondrial matrix | C | chlo: 10, cyto: 2, mito: 2 |
| Py01658 | Golgi apparatus. Nucleus. | mitochondrial matrix\chloroplast stroma\ nucleus | C | chlo: 13 |

| PyHAT | Plant-mPLoc | PSORT Prediction (plant model) | predalgo | wolf PSOR (plant) |
| --- | --- | --- | --- | --- |
| Py02283 | Nucleus. | cytoplasm |  | chlo: 13 |
| Py03649 | Nucleus. | microbody (peroxisome)\ nucleus |  | chlo: 4, cyto: 4, Pero: 3, nucl: 2 |
| Py04694 | Cytoplasm.Peroxisome | endoplasmic reticulum |  | cyto: 12, chlo: 2 |
| Py09866 | Cytoplasm. Nucleus. | mitochondrial matrix |  | cyto: 12, chlo: 2 |
| Py00356 | Nucleus. | chloroplast stroma\nucleus |  | nucl: 12, chlo: 1 |
| Py07125 | Nucleus. | nucleus |  | nucl: 14 |
